# Quantification of mobile charge carrier yield and transport lengths in ultrathin film light-trapping ZnFe_2_O_4_ photoanodes†

**DOI:** 10.1039/d4ta05448b

**Published:** 2024-12-18

**Authors:** Kumaraswamy Miriyala, Sa'ar Shor Peled, Dino Klotz, Daniel A. Grave

**Affiliations:** a Department of Materials Engineering, Ben-Gurion University of the Negev Beer Sheva 8410500 Israel dgrave@bgu.ac.il; b Ilse Katz Institute for Nanoscale Science and Technology, Ben-Gurion University of the Negev Beer Sheva 8410500 Israel; c International Institute for Carbon-Neutral Energy Research (I2CNER), Kyushu University Fukuoka Japan

## Abstract

Zinc ferrite (ZnFe_2_O_4_, ZFO) has gained attention as a candidate material for photoelectrochemical water oxidation. However, champion devices have achieved photocurrents far below that predicted by its bandgap energy. Herein, strong optical interference is employed in compact ultrathin film (8–14 nm) Ti-doped ZFO films deposited on specular back reflectors to boost photoanode performance through enhanced light trapping, resulting in a roughly fourfold improvement in absorption as compared to films deposited on transparent substrates. The spatial charge carrier collection profile and wavelength-dependent photogeneration yield of mobile charge carriers was then extracted *via* spatial collection efficiency analysis based on optical and external quantum efficiency measurements. We demonstrate that despite the enhanced performance enabled by the light trapping structure, substantial recombination occurs for thin film ZFO photoanodes even within the space charge region of an ultrathin film photoanode. Furthermore, the excitation-wavelength-dependent yield of mobile charge carriers in ZFO is shown to be less than unity across the visible spectrum, ultimately limiting the attainable photocurrent density. These results explain the underperformance of ZFO as a photoanode material and suggest that reduction of the mobile charge carrier yield due to the existence of ligand field states is a dominant loss mechanism for metal-oxides containing Fe metal centers with open d-shell configuration.

## Abstract 2

Resonant light trapping is used to enhance the performance of Ti-doped ZnFe_2_O_4_ (ZFO) photoanodes. Spatial collection efficiency analysis shows that non-unity photogeneration yield of mobile charge carriers limits the performance of ZFO.

## Graphical abstract

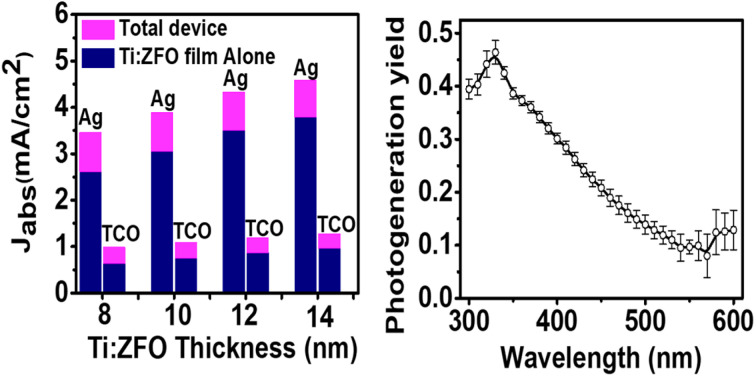

## 1. Introduction

Photoelectrochemical (PEC) hydrogen generation uses sunlight to directly split water through use of a semiconducting photoabsorber.^1^ Despite the promise of this approach for renewable energy production, an ideal photoelectrode material has not yet been developed. Among the most widely studied photoanode materials for water oxidation are TiO_2_ and BiVO_4_ (ref. 2–5) which have reached high internal quantum efficiencies, but their bandgaps are too large for practical water splitting applications. Hematite (α-Fe_2_O_3_) with a suitable bandgap of ∼2 eV has been widely studied, but the best performing photoanodes only reach about half the theoretical maximum photocurrent density.^6–9^ Recently, this performance limitation has been attributed to ligand field states which reduce the yield of photogenerated mobile carriers, thereby ultimately limiting the attainable photocurrent under AM 1.5 G conditions to roughly half that predicted based on the bandgap.^10–12^ As this may be an intrinsic limitation to hematite photoanodes, there is interest to investigate the PEC properties of other stable, abundant metal-oxide materials with appropriate light absorption capabilities.

ZnFe_2_O_4_ (ZFO) has garnered interest as a photoanode material due to several advantageous properties including its ∼2 eV bandgap, stability in aqueous solution, and low cost.^13–19^ However, the small absorption coefficient of ZFO^20^ coupled with the reportedly poor charge transport properties results in substantial recombination of photogenerated charge carriers in highly absorbing planar thick film photoanodes. To overcome the trade-off between charge carrier transport length and the absorption depth of light, nanostructured photoanodes have been developed to improve photoanode performance. Such developments have boosted the maximal achieved photocurrent densities to roughly ∼1 mA cm^−2^ under AM 1.5 G solar illumination, yet far from the theoretical maximum of roughly ∼12 mA cm^−2^.^19,21^

We introduce an alternate approach to improve ZFO photoanode performance by employing light trapping in ultrathin films deposited on specular back reflectors. Strong interference can enhance broadband optical absorption in ultrathin planar layers without need for nano structuring.^22,23^ Here, we grow ultrathin (8–14 nm thick) Ti-doped ZnFe_2_O_4_ (Ti:ZFO) films on epitaxial silver thin films coated with a Nb-doped SnO_2_ (NTO) layer serving as an electron selective contact. We show that resonant light trapping enhances absorption in ultrathin film ZFO photoanodes by roughly four times, leading to enhanced absorption and photocurrent density compared to films of similar thickness deposited on transparent conducting oxides (TCOs).

Despite significant improvement of the light-trapping photoanodes over conventional planar thin films deposited on TCOs, the photoconversion efficiency remains surprisingly low. We identify, through spatial collection efficiency analysis, a charge transport length of ∼5 nm for minority carriers within the depletion region of the ultrathin ZFO films leading to substantial recombination of photogenerated carriers. In addition, we extract the yield of photogenerated mobile charge carriers in ZFO and show that it is significantly less than unity across the visible spectrum, ultimately limiting the performance of ZFO photoelectrodes.

## 2. Experimental section

### 2.1 Target and film preparation

Initially, a 1 cation % Ti doped ZFO (hereafter referred as Ti:ZFO) and a 1 cation % Nb doped SnO_2_ (hereafter referred as NTO) targets were prepared by solid state reaction route using commercially available high purity (≥99.99%) powders. For Ti:ZFO target preparation, stoichiometric amounts of ZnO, Fe_2_O_3_, and TiO_2_ powders (NOAH Technologies Corp.) were mixed thoroughly and calcined at 900 °C for 2 h to get phase pure Ti:ZFO powder. Later the calcined powder was pelletized and sintered at 1200 °C for 8 h. The obtained target density is around 95% of its theoretical density. The NTO target was prepared by spark plasma sintering (Prof. Shmuel Hayun's lab, Department of Materials Engineering, Ben-Gurion University, Israel). The fine grinded Nb_2_O_5_ (Acros Organics) and SnO_2_ powder (Alfa Aesar) mixture was taken into a 20 mm graphite die and sintered at 1075 °C for 15 minutes. Later these targets were used to grow NTO and Ti:ZFO thin films. Prior to these thin film's growth, a thick silver (Ag∼170 nm) film was coated on a freshly cleaved mica substrate (Ted Pella, Inc.) by DC magnetron sputtering. The base pressure of the chamber was below 5 × 10^−6^ Torr and the substrate temperature setpoint was 300 °C. Later the chamber pressure was raised to 5 mTorr using Ar gas and the DC power increased to 250 W to initiate the Ag deposition on the mica substrate. Pulsed laser deposition (PVD Products, Nano-PLD-1000) was used to fabricate the Ti:ZFO and NTO layers on the Ag/mica substrate. The depositions were carried out at a substrate setpoint temperature of 500 °C and an oxygen partial pressure of 25 and 10 mTorr for Ti:ZFO and NTO films, respectively. A KrF excimer laser (Coherent, COMPex 102F) operating at 248 nm was used with an energy density of 1.5 J cm^−2^ per pulse and repetition rate of 3 Hz. The target to substrate distance was maintained at 7 cm throughout the deposition.

### 2.2 Materials characterization

High-resolution X-ray diffraction (HR-XRD) was used to identify the phase purity and crystallographic orientation of the films. The XRD patterns were collected using Cu Kα radiation (*λ* = 1.5406 Å), operating at 45 kV and 40 mA (Panalytical: Empyrean III) with parallel beam optics accompanied with a 2-bounce monochromator. Standard *θ*–2*θ* measurements were performed to identify the out-of-plane crystallographic orientation and off-axis phi scans in skew symmetric geometry were performed to probe the in-plane alignment. Atomic force microscopy (AFM) (MFP-3D-Bio; Asylum Research, Oxford instrument) was used in tapping mode configuration to measure the surface roughness of the films. High-resolution transmission electron microscopy (JEOL JEM-2100F, operated at 200 keV) studies were performed to analyze the microstructural features and individual film thickness in cross-sectional view. The specular reflectance spectra (*R*) were measured using Agilent CARY5000 UV-vis spectrometer system with UMA accessory. Further, the optical constants of the Ti:ZFO films were measured by using a spectroscopic ellipsometer (J. A. Woollam, Model: M-2000UI) in the 200–1000 nm wavelength range and at an incident angles of 65,70 and 75°.

### 2.3 Photoelectrochemical characterization

The photoelectrochemical measurements were performed under solar simulated light (Sciencetech AAA solar simulator) in 1 M NaOH alkaline electrolyte in a custom built photoelectrochemical cell. A 3-electrode configuration was used with an Hg/HgO reference electrode and platinum wire as a counter electrode. Incident photon conversion efficiency (IPCE) measurements were performed using a ScienceTech PTS-2 system with a spectral range of 250 to 620 nm. Intensity modulated photocurrent spectroscopy (IMPS) measurements were performed using Zennium pro electrochemical workstation (Zahner) equipped with a white LED light source outputting an intensity of 100 mW cm^−2^. A sinusoidal modulated light intensity was applied with a modulation depth of 30% in the frequency range of 10 kHz to 300 mHz. Electrochemical impedance spectroscopy (EIS) measurements were conducted using a sinusoidal signal with 10 mV amplitude over a frequency range of 0.1 Hz–100 kHz under solar simulated light.

## 3. Results and discussion

### 3.1 Crystallographic and structural characterization

**Fig. 1:**
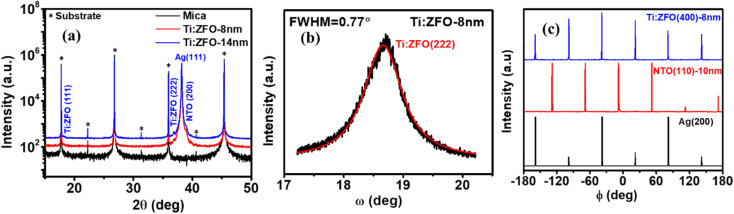
(a) HR-XRD *θ*–2*θ* patterns for 8 and 14 nm Ti:ZFO/NTO/Ag films (b) rocking curve analysis of 8 nm Ti:ZFO film (c) off-axis phi scans for Ti:ZFO(400), NTO(110) and Ag(200) peaks.

Ti:ZFO/NTO/Ag film stacks were grown on mica substrates by a combination of pulsed laser deposition and DC magnetron sputtering as described in the methods section. The Ti:ZFO film thickness was varied between 8 and 14 nm. Ti-doping was incorporated into the photoelectrodes as it has recently been shown to improve the properties of ZnFe_2_O_4_ photoelectrodes.^24–26^ The NTO underlayer was employed as an electron selective contact, which blocks backward hole injection to the Ag substrate due to the large valence band offset between SnO_2_ and ZnFe_2_O_4_.^27^ High-resolution X-ray diffraction (HR-XRD) measurements were performed to confirm the heteroepitaxial growth of the Ti: ZFO thin films. Fig. 1a displays the HR-XRD *θ*–2*θ* scans of the 8 and 14 nm Ti:ZFO films. Diffraction is only observed from a single set of planes for every layer, with the out-of-plane orientation relationship given as Ti:ZFO(111)/NTO(200)/Ag(111). Fig. 1b shows a rocking curve measurement of the Ti:ZFO (222) symmetric reflection to identify the misorientation of the domains in the out-of-plane direction. The measured full width half maximum of the (222) reflection for the 8 nm film was 0.77°, which suggests the presence of some out-of-plane mosaicity in the Ti:ZFO layer. To check the in-plane alignment, off-axis phi scans in skew-symmetric geometry were performed for the 8 nm Ti:ZFO (400), NTO (110), and Ag (200) reflections as shown in Fig. 1c. For all layers, sharp and distinct peaks are observed, confirming the heteroepitaxial growth of the Ti:ZFO/NTO/Ag film stack along the in-plane direction. We observed six reflections in the Ag (200) *ϕ* scan rather than three, indicative of an additional rotational domain rotated about the [111] axis. This type of rotational twining is commonly observed in Ag films deposited on mica.^23^ Following growth of the Ag layer, the NTO (110) *ϕ* scan also shows six-fold symmetry instead of two, suggesting the existence of three different in-plane rotational domains. Finally, the Ti:ZFO (400) *ϕ* scan also shows sixfold symmetry instead of expected threefold symmetry, indicating the existence of twinned in-plane rotational domains.

**Fig. 2:**
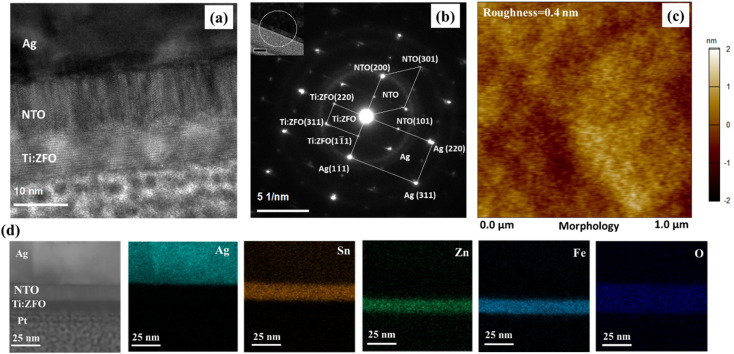
(a) HR-TEM image for 8 nm Ti:ZFO/NTO/Ag interface (b) SAED pattern for Ag, NTO and Ti:ZFO measured at <1̄12>_Ag_ zone axis (c) AFM morphology of 8 nm Ti:ZFO/NTO/Ag film (d) HAADF image of Ti:ZFO/NTO/Ag interface along with EDS mapping of various elements across the interface.

Fig. 2a displays a high-resolution TEM (HR-TEM) image of the 8 nm thick Ti:ZFO and 10 nm thick NTO thin films grown on Ag/Mica in cross sectional view, revealing the clear interface between the layers. Distinct microstructural differences are observed between the two layers. While the NTO displays a nanocolumnar morphology as a result of the growth of the three in-plane domains, the Ti:ZFO film showed a dense microstructure, with no boundaries observed within the cross sectional image. A selected area electron diffraction (SAED) pattern measured across the complete stack (Ti:ZFO/NTO/Ag) is shown in Fig. 2b. The NTO layer showed streaks in its diffraction pattern resulting from its nanocolumnar morphology while the Ag, Ti:ZFO layers displayed circular diffraction spots indicative of single crystallinity. Further analysis of the SAED pattern confirms the heteroepitaxial growth of NTO and Ti:ZFO layers on the Ag/Mica substrate. The obtained crystallographic orientation relationship between the layers is as follows: Ag(111)/NTO(2000/Ti:ZFO(111)) and Ag[1̄12]‖NTO[02̄0]‖Ti:ZFO[1̄12]. In addition, the AFM image of Ti:ZFO film (8 nm) shown in Fig. 2c displays a smooth and crack free surface with 0.4 nm root mean square (RMS) roughness over a 1 × 1 μm^2^ area. Cross sectional TEM-HAADF micrographs of the thin film stack are shown in Fig. 2d along with energy dispersive X-ray spectroscopy (EDS) elemental maps, showing clear boundaries of the layers.

### 3.2 Optical characterization

**Fig. 3:**
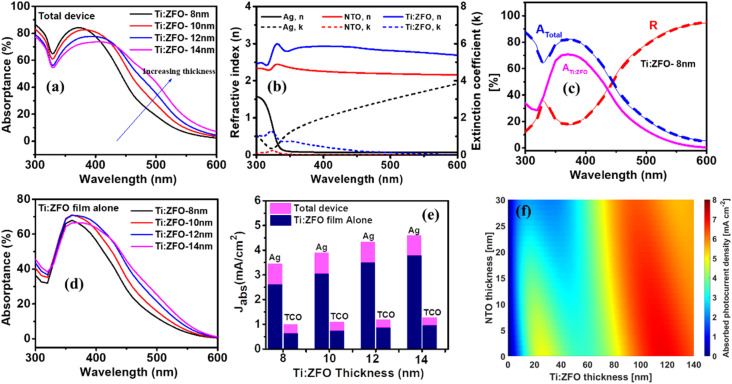
(a) Absorptance of total device (Ti:ZFO/NTO/Ag/Mica) including parasitic absorption in the Ag and NTO layers measured by UV-vis spectrophotometry for the 8, 10, 12, and 14 nm films (b) optical constants extracted from spectroscopic ellipsometry (c) simulated and measured reflectance and absorptance of the total device for the 8 nm film. Also shown is the calculated absorptance in the Ti:ZFO active layer alone. (d) Calculated absorptance in the Ti:ZFO active layer alone for 8, 10, 12, and 14 nm films. (e) Calculated *J*_abs_ in the Ti:ZFO films compared to *J*_abs_ in films of similar thickness deposited on a transparent conducting oxide. (f) Calculated *J*_abs_ map in the Ti:ZFO layer alone as a function of NTO and Ti:ZFO thickness.

The specular reflectance of the photoanode film stacks was measured using UV-vis spectrophotometry, allowing for calculation of the absorptance (Fig. 3a) in the device *via* the following relationship: *A* = 100−*R*−*T*, where *R* and *T* are the measured reflectance and transmittance, respectively. We note that this calculation is for the total absorptance in the full device and includes parasitic absorption in the Ag and NTO layers in addition to absorption in the photoactive Ti:ZFO layer. To calculate the absorptance in each individual layer, we combine spectroscopic ellipsometry measurements and modelling (see Fig. S1†) with transfer matrix method simulations. Initially, spectroscopic ellipsometry measurements were performed on the films to extract the layer optical constants which are shown in Fig. 3b. Details of the spectroscopic ellipsometry modelling is provided in the ESI.† Next, the optical constants were used to calculate the specular reflectance *via* transfer matrix method simulations.^22,28^Fig. 3c displays the simulated and measured specular reflectance of the 8 nm thick Ti:ZFO film. Good agreement between the simulated and measured values is shown, validating the optical model and allowing for calculation of the absorptance in the Ti:ZFO film alone. The simulated and measured spectrophotometry data of the additional 10, 12, and 14 nm thick films are shown in Fig. S2a–c.† All simulations exhibit good agreement to the measured data, allowing for comparison between the various photoanode stacks. Fig. 3d shows the absorptance in the Ti:ZFO photo absorber alone for various film thicknesses. Comparing Fig. 3a and d shows the importance of extracting the absorptance in the Ti:ZFO layer alone through simulation rather than relying on UV-vis spectrophotometer measurements of the full device. The decline in absorptance in the total device (Fig. 3a) in the wavelength range between ∼300–430 nm as the film thickness increases from 8 to 14 nm can be attributed to reduced parasitic absorption in the NTO and Ag layers (shown in Fig. S2d†). We note that as compared to hematite films deposited on silver back reflectors,^23^ the parasitic absorptance in Ti:ZFO based devices is higher due to the smaller absorption coefficient of ZFO.

The absorbed photon flux (*J*_abs_) expressed as a current density within the Ti:ZFO photo absorber or full stack under solar AM 1.5 G illumination can be calculated using the following expression: 1
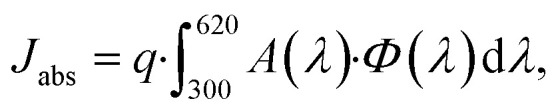


where *q* is the electron charge, *A*(*λ*) refers to the absorption in the Ti:ZFO film alone or in the film stack, and *Φ* is the photon flux defined by the AM 1.5 G solar spectrum. *J*_abs_ for the different films deposited in this study is shown in Fig. 3e and compared to simulations made for the same thickness of Ti:ZFO deposited on a transparent conducting oxide (TCO) current collector. As can be observed, due the strong interference effects enabled by the silver reflector, the absorption is highly sensitive to small changes in film thickness. From only a 6 nm change in Ti:ZFO film thickness from 8 to 14 nm, the absorption is substantially enhanced with the value of *J*_abs_ increasing from 2.61 to 3.78 mA cm^−2^ in the Ti:ZFO layer alone. In comparison, the *J*_abs_ values for the Ti:ZFO films deposited on a TCO range from only 0.63 to 0.96 mA cm^−2^. This shows that the strong interference effects enabled by the silver back reflector lead to a nearly fourfold increase in *J*_abs_.

To further examine the effect of film thickness on the Ti:ZFO films, a *J*_abs_ map was simulated across a range of NTO and Ti:ZFO thicknesses as shown in Fig. 3f. For a given thickness of NTO, it is possible to observe two clear resonance modes with local maxima for *J*_abs_. For Ti:ZFO without any underlayer, these maxima occur at 26 and 116 nm film thicknesses corresponding to *J*_abs_ of 4.86 and 7.08 mA cm^−2^, respectively. For comparison, hematite ultrathin films can reach up to 10 mA cm^−2^ in the film alone at the first resonance mode of roughly ∼20 nm due to the higher absorption coefficient.^23^ By comparing *J*_abs_ of the full stack (shown in Fig. S3†) and *J*_abs_ of the Ti:ZFO layer, it can be observed that at the first resonance mode, there is roughly 30% parasitic absorption due to the low absorption coefficient of ZFO. At the second resonance mode, this parasitic absorptance is lessened to ∼10%, however at the cost of a film thickness much larger than the transport length, which will lead to significant carrier recombination under device operation. These results suggests that the low absorption coefficient of ZFO is a challenge towards its implementation in resonant light trapping structures.

### 3.3 Photoelectrochemical characterization

**Fig. 4:**
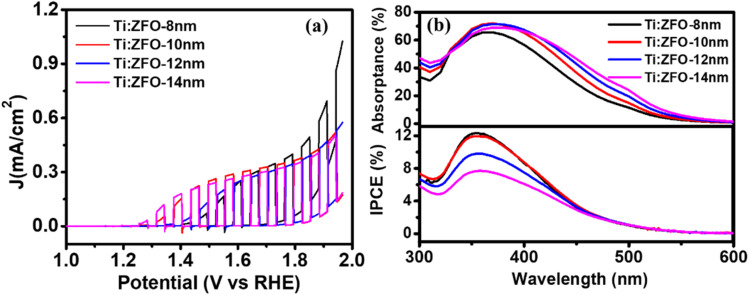
(a) Linear voltammograms of Ti:ZFO films measured under solar simulated light in 1 M NaOH solution. (b) Comparison of calculated absorptance spectra in Ti:ZFO layer alone and IPCE measured at 1.76 V_RHE_.

Turning to the photoelectrochemical properties of the Ti:ZFO photoanodes, linear sweep voltammograms measured in 1 M NaOH solution under solar simulated light (AM 1.5 G) are shown in Fig. 4a. It can be seen that the thinner 8 and 10 nm films show slightly higher photocurrents than the thicker films, reaching saturation photocurrents of roughly ∼340 μA cm^−2^ at high potential. To our knowledge, this is the highest reported saturation photocurrent density for compact planar ZFO thin films without a nanostructured morphology reported in the literature. To further demonstrate the advantage of resonance light trapping, we measured the linear voltammograms (Fig. S4†) of epitaxial Ti:ZFO films of the same thickness deposited on transparent current collectors (Nb:SnO_2_ coated sapphire). The obtained saturation photocurrent values are in the range of 80–90 μA cm^−2^, approximately four times lower in comparison with the films grown on the Ag substrate. The improvement in photocurrent is consistent with the increase in absorbed photocurrent density (Fig. 3e) resulting from the resonant light trapping effect.

Incident photon conversion efficiency (IPCE) measurements were then performed at applied potential of 1.76 V *vs.* RHE to ensure all photoanodes were at the saturation photocurrent and surface recombination was minimized. As shown in Fig. 4b, the highest IPCE in the ∼300–475 nm spectral range was observed for the 8 and 10 nm thin films, followed by a significant reduction of the IPCE in this range for the 12 and 14 nm thick films. Interestingly, above 475 nm, no difference in the IPCE was observed for the films, despite a nearly twofold enhancement in absorptance in the thicker films. To confirm that surface recombination is negligible at 1.76 V_RHE_, we performed intensity modulated photocurrent spectroscopy (IMPS) measurements on all the films (Fig. S5†). IMPS spectra were collected at three different potentials: 1.16 V_RHE_ (below onset), 1.46 V_RHE_ (near onset region), and 1.76 V_RHE_. (plateau region where the IPCE was measured). With increasing applied potential, the diameter of the bottom semi-circle increases, indicative of increasing hole flux density to the surface. Furthermore, the diameter of the top semi-circle decreases with increasing potential, indicating a decrease in surface recombination. At a potential of 1.76 V_RHE_ where the IPCE measurements were performed, the top semi-circle nearly disappears indicating that surface recombination is minimal. To assess the charge transfer (*η*_t_) and charge separation efficiencies (*η*_cs_) quantitively, the IMPS spectra were fitted to an equivalent circuit model.^29^ The high frequency intercept with the real axis (HFI, representing the hole current density), and low frequency intercept with the real axis (LFI, representing the device photocurrent density) were extracted from the model and *η*_t_ was calculated as:
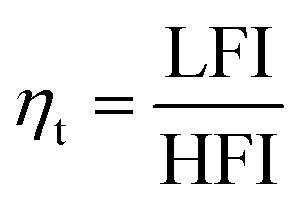


The obtained *η*_t_ values for the Ti:ZFO films of 8–14 nm thickness was calculated to be 99% at a potential of 1.76 V_RHE_ for all films. Therefore, the effect of surface recombination is negligible at this potential and the measured IPCE spectra represent bulk transport properties. The obtained charge transfer efficiency (*η*_t_) and charge separation efficiency (*η*_cs_) for all the samples are listed in Table S2 and S3.† Due to the small transport length of ∼5 nm that will be discussed below, the separation efficiency decreases with increasing film thickness.

Fig. S6† shows the photocurrent density obtained from the integration of the IPCE spectra over the AM 1.5 G solar spectrum. Comparison with the photocurrent (*J*_photo_) obtained from the LSV measurements at 1.76 V_RHE_ are shown in Table S4.† In addition, stability tests and EIS measurements for all the samples are shown in Fig. S7 and S8.† The EIS measurements further showed that charge transfer resistance decreases with increasing applied potential.

Before the introduction of spatial collection efficiency analysis below which will allow quantitative analysis of the losses in the photoanode, it is helpful to qualitatively examine the relationship between light intensity within the layer and the IPCE results. We focus on two wavelengths, 350 nm, where there is a peak in the IPCE spectra and substantial difference in the measured values between the different films, and 500 nm, where the IPCE is similar for all the films. The light intensity as a function of wavelength and depth in the layer was calculated and plotted in Fig. S9.† It can be observed that there is higher intensity of 350 nm light absorbed closer to the surface in the thinner films. As the film thickness increases, the absorbed light intensity is more gradual along the film thickness for 350 nm illumination. This results in a higher IPCE at 350 nm for the thinner films despite the lower overall absorptance at this wavelength. The IPCE and light absorption profiles for 500 nm wavelength show different behavior. A negligible increase in IPCE is observed despite the roughly twofold increase in absorptance at this wavelength, a surprising result given only a 6 nm difference in film thickness. The light intensity for the 14 nm thick film is higher throughout the film thickness for 530 nm illumination, precluding transport of charge carriers as the sole reason for this behavior.

### 3.4 Spatial collection efficiency analysis

To further investigate the PEC performance in these films, we employ spatial collection efficiency analysis, a non-destructive characterization technique that can be used to analyze losses in photovoltaic and photoelectrochemical cells based on IPCE measurements and optical absorption profiles.^30–33^ The IPCE can be described by the following integral:2
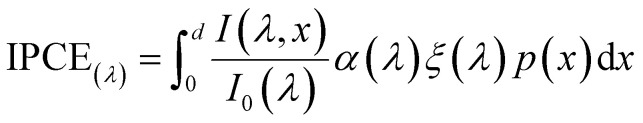
where 
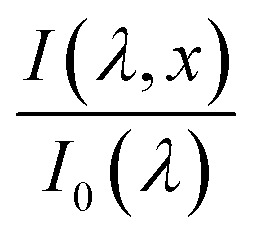
 is the wavelength-dependent photon flux at distance *x* from the surface divided by the incident flux at the surface, *α*(*λ*) is the absorption coefficient, *p*(*x*) is the spatial collection efficiency, or the probability of a hole generated at depth *x* within the film will reach the surface and contribute to the photocurrent, and *ξ*_(*λ*)_ is the photogeneration yield, defined as the probability that a photon of wavelength *λ* will generate a mobile electron–hole pair. The IPCE is measured while the incremental absorption in the layer given by the term 
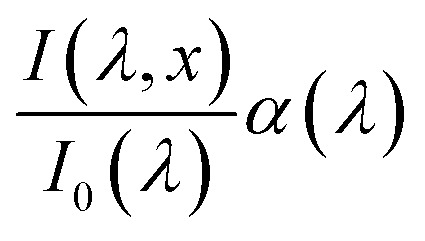
 is calculated using TMM simulations, which are verified by UV-vis spectrophotometry measurements as shown earlier in Fig. 3. The remaining unknowns, *p*(*x*) and *ξ*(*λ*), must be extracted which presents a numerical challenge that requires some *a priori* assumptions.^11,32^

Here, we introduce an algorithm that extracts *p*(*x*) and *ξ*_(*λ*)_, and reconstructs the experimental IPCE spectra based minimal assumptions and on only one or two free parameters describing charge transport lengths of the minority carriers in the space charge or quasi-neutral region. The spatial collection efficiency (SCE, *p*_(*x*)_) is written as a piecewise function:^11^3
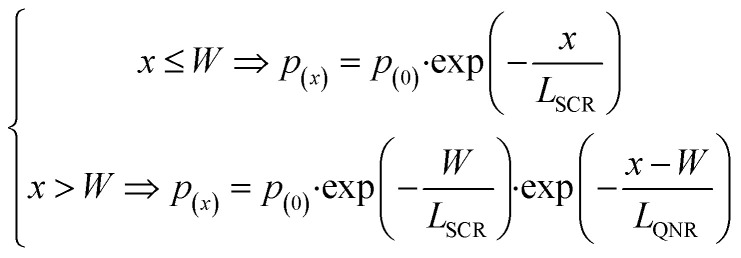
where *p*_(0)_ is the probability of hole injection at the surface, *W* is the depletion width, *L*_SCR_ is the minority carrier transport length in the space-charge (depletion) region, and *L*_QNR_ is the minority carrier transport length in the quasi-neutral region.

**Fig. 5:**
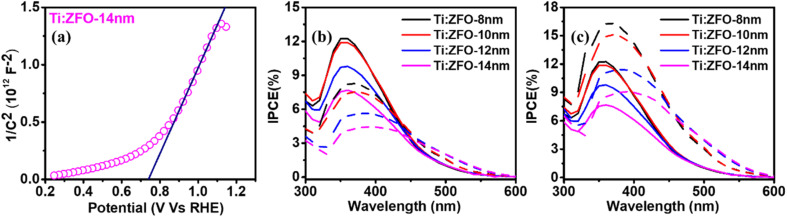
(a) Mott–Schottky plot of 14 nm thick Ti:ZFO film. Measured (solid) and simulated (dashed) IPCE according to eqn (2) assuming a unity value of *ξ*_(*λ*)_ and *L*_SCR_ values of (b) 1 nm and (c) 2 nm.

To reduce the number of free variables and *a priori* assumptions, the space charge region width in the ultrathin Ti:ZFO films was extracted by capacitance-potential measurements and Mott–Schottky analysis. The Mott–Schottky plot of the 14 nm thin film is shown in Fig. 5a. The corresponding space charge layer width, *W*, at 1.76 V *vs.* RHE was calculated *via* the following formula:4
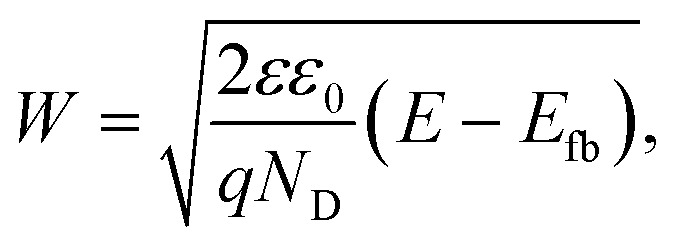
where *ε* is the dielectric constant of ZFO, *ε*_0_ is the vacuum permittivity, *E*_fb_ is the flat band potential, and *N*_D_ is the donor density. Assuming a previously reported value of the dielectric constant of 100,^18,34^ we extract *N*_D_ = 3.45 ± 0.5 × 10^19^ cm^−3^ from the slope of the Mott–Schottky curve, and *E*_fb_ = 0.74 ± 0.01 V *vs.* RHE from the *x*-axis intercept, corresponding to a space charge layer width of 18.2 ± 1.2 nm at 1.76 V_RHE_, larger than the film thicknesses of 14 nm, suggesting that the film is fully depleted. The Mott–Schottky plots for the 8, 10, and 12 nm films are shown in Fig. S10† along with the values of *N*_D_, *W* and *E*_fb_ listed in Table S5.† For all the films, the extracted depletion width is greater than the film thickness at 1.76 V_RHE_. Therefore, it is assumed that all the films are fully depleted and *p*(*x*) can be described by a homogenous region with a single transport length, as described in the first part of the piecewise function for *x* ≤ *W*. The value for *p*0 was fixed as the charge transfer efficiency extracted from the IMPS measurement.

The algorithm initially reconstructs the IPCE by integrating the product of *A*_(*x*,*λ*)_*p*_(*x*)_ over the film thickness for unique values of *L*_SCR_, assuming a unity value for *ξ*_(*λ*)_. As shown in Fig. 5b, calculation of the IPCE spectrum assuming unity *ξ*_(*λ*)_ with a conservative value of *L*_SCR_ = 1 nm significantly underestimates the experimental IPCE in the low wavelength (<450 nm) region while overestimating the IPCE in the long wavelength (>450 nm) region. When *L*_SCR_ is increased to a value of 2 nm, as shown in Fig. 5c, the simulated IPCE overestimates the measured IPCE throughout nearly the entire wavelength range for all the samples. These results suggest that the IPCE spectrum of the Ti:ZFO films cannot be simulated assuming a unity value for *ξ*_(*λ*)_.

To account for the non-unity *ξ*_(*λ*)_, the next step in the algorithm is to divide the measured IPCE spectrum with the calculated IPCE spectrum based on unity *ξ*_(*λ*)_, which gives an approximation for the corrected value of *ξ*_(*λ*)_. Given that *ξ*_(*λ*)_ is an intrinsic material property, and that there is a minor spread in film thickness, it can be assumed that the *ξ*_(*λ*)_is similar for all films. This is supported by ellipsometry analysis where the same absorption coefficient was extracted for all the thin films, demonstrating that there is no significant change in the optical properties within this small thickness range. The algorithm then iterates this calculation for a range of *L*_SCR_ values, calculating the variance between the maxima of the *ξ*_(*λ*)_ spectra for the different films for each *L*_SCR_ value.

**Fig. 6:**
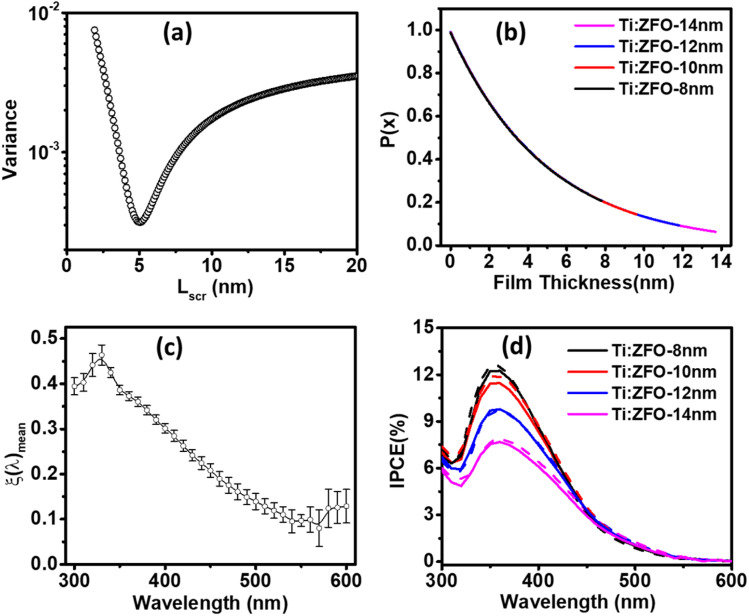
(a) Variance between maximal values of *ξ*_(*λ*)_ for different Ti:ZFO films as a function of *L*_SCR_. (b) *p*(*x*) profile based on *L*_SCR_ of 5 nm for Ti:ZFO films. (c) Mean *ξ*_(*λ*)_ spectra extracted from algorithm. (d) Measured (solid) and simulated (dashed) IPCE according to eqn (2) assuming *p*(*x*) profile and mean *ξ*_(*λ*)_ spectrum shown in Fig. 6b and c, respectively.

Fig. 6a shows the variance between the maximal values of the *ξ*_(*λ*)_ spectra of the films plotted for values of *L*_SCR_ ranging from 1–20 nm, with minimum variance found for *L*_SCR_ = 5 nm. Inserting this value for *L*_SCR_ in eqn (3) yields the *p*(*x*) profile observed in Fig. 6b. Multiplying the product of the *p*(*x*) profile of each film with the corresponding film absorption profile, 
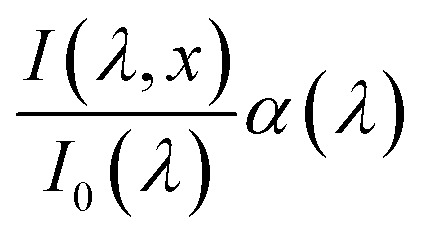
, and the mean value of *ξ*_(*λ*)_ shown in Fig. 6c, according to eqn (2) allows for calculation of the IPCE spectra. As shown in Fig. 6d, good agreement with the experimental IPCE spectra was observed for all the films. We emphasize these values are obtained using the same value of *L*_SCR_ and the same *ξ*_(*λ*)_ spectrum for all films, which accurately describe the IPCE behavior of all four films.

The implication of the *ξ*_(*λ*)_ spectrum, peaking at ∼40% in the UV region of the solar spectrum and reaching as low as 10% in the green region suggests that the yield of mobile charge carriers in ZFO substantially limits the attainable photocurrent in the material. Indeed, the photogeneration yield spectrum appears to be consistent with the highest reported IPCEs for ZFO previously reported in the literature,^21^ suggesting that techniques such as nanostructuring may not be able to further improve ZFO photoanode performance. Furthermore, the photogeneration yield spectrum appears to have similar shape to that recently found for ultrathin film hematite photoanodes,^10^ implying that this loss mechanism may be intrinsic to metal-oxides which possess Fe^3+^ cations with d^5^ electronic configuration.

## 4. Conclusions

We demonstrate that deposition of ultrathin films on specular back reflectors can be used to resonantly trap light in Ti:ZFO, thereby substantially improving absorption and PEC performance as compared to films deposited on TCOs. Despite these improvements, spatial collection efficiency analysis shows that the minority charge carrier transport properties of ZFO are severely limited, resulting in substantial recombination even in the space charge region of ultrathin films, thereby presenting a challenge for envisioned practical device applications. Secondly, a non-unity photogeneration yield of mobile charge carriers limits the attainable photocurrent to much lower than predicted by the material's band gap. The similarity of the extracted photogeneration yield spectrum compared to that extracted for hematite photoanodes suggests that this limitation may be intrinsic to metal-oxide photo absorbers with Fe^3+^ cations in d^5^ electron configuration. Based on these results, future efforts to improve the PEC performance of ZFO should focus on how to increase the yield of mobile charge carriers.

## Data availability

The data supporting this article have been included as part of the ESI.†

## Conflicts of interest

There are no conflicts of interest to declare.

## Supplementary Material

TA-013-D4TA05448B-s001

## References

[cit1] Fujishima A., Honda K. (1972). Nature.

[cit2] Yu Z., Liu H., Zhu M., Li Y., Li W., Yu Z., Liu H., Zhu M., Li Y., Li W. (2021). Small.

[cit3] Wang G., Wang H., Ling Y., Tang Y., Yang X., Fitzmorris R. C., Wang C., Zhang J. Z., Li Y. (2011). Nano Lett..

[cit4] Qin Q., Cai Q., Li J., Jian C., Hong W., Liu W. (2019). Sol. RRL.

[cit5] Zhou C., Sanders-Bellis Z., Smart T. J., Zhang W., Zhang L., Ping Y., Liu M. (2020). Chem. Mater..

[cit6] Park J., Yoon K. Y., Ghule B. G., Kim H., Jang J. H. (2024). ACS Energy Lett..

[cit7] Guo X., Wang L., Tan Y. (2015). Nano Energy.

[cit8] Li J., Qiu Y., Wei Z., Lin Q., Zhang Q., Yan K., Chen H., Xiao S., Fan Z., Yang S. (2014). Energy Environ. Sci..

[cit9] Qiu Y., Leung S. F., Zhang Q., Hua B., Lin Q., Wei Z., Tsui K. H., Zhang Y., Yang S., Fan Z. (2014). Nano Lett..

[cit10] Grave D. A., Ellis D. S., Piekner Y., Kölbach M., Dotan H., Kay A., Schnell P., van de Krol R., Abdi F. F., Friedrich D., Rothschild A. (2021). Nat. Mater..

[cit11] Piekner Y., Ellis D. S., Grave D. A. (2021). Anton Tsyganok and Avner Rothschild. Energy Environ. Sci..

[cit12] Hayes D., Hadt R. G., Emery J. D., Cordones A. A., Martinson A. B. F., Shelby M. L., Fransted K. A., Dahlberg P. D., Hong J., Zhang X., Kong Q., Schoenlein R. W., Chen L. X. (2016). Energy Environ. Sci..

[cit13] Bohra M., Alman V., Arras R. (2021). Nanomaterials.

[cit14] Polo A., Boudoire F., Lhermitte C. R., Liu Y., Guijarro N., Dozzi M. V., Selli E., Sivula K. (2021). J. Mater. Chem. A.

[cit15] Henning R. A., Uredat P., Simon C., Bloesser A., Cop P., Elm M. T., Marschall R. (2019). J. Phys. Chem. C.

[cit16] Hufnagel A. G., Peters K., Müller A., Scheu C., Fattakhova-Rohlfi ng D., Bein T., Hufnagel A. G., Peters K., Fattakhova-Rohlfi ng D., Bein T., Müller A., Scheu C. (2016). Adv. Funct. Mater..

[cit17] Zheng X. L., Dinh C. T., de Arquer F. P. G., Zhang B., Liu M., Voznyy O., Li Y. Y., Knight G., Hoogland S., Lu Z. H., Du X. W., Sargent E. H. (2016). Small.

[cit18] Tahir A. A., Wijayantha K. G. U. (2010). J. Photochem. Photobiol., A.

[cit19] Zhu X., Guijarro N., Liu Y., Schouwink P., Wells R. A., Le Formal F., Sun S., Gao C., Sivula K., Zhu X., Guijarro N., Liu Y., Wells R. A., Le Formal F., Sivula K., Sun S., Gao C., Schouwink P. (2018). Adv. Mater..

[cit20] Tan R., Jeong Y. J., Li Q., Kang M., Cho I. S. (2023). J. Adv. Ceram..

[cit21] Kim J. H., Choi Y., Kim J. H., Kim J., Kim Y. K., Kim J. K., Lee J. S., Kim J. H., Kim Y. K., Lee J. S., Choi I. Y., Kim J., Kim J. K. (2021). Small.

[cit22] Dotan H., Kfir O., Sharlin E., Blank O., Gross M., Dumchin I., Ankonina G., Rothschild A. (2013). Nat. Mater..

[cit23] Shor Peled S., Miriyala K., Rashkovskiy A., Fishov R., Gelberg V., Pelleg J., Grave D. A. (2023). ACS Appl. Mater. Interfaces.

[cit24] Shriqui Y., Rashkovskiy A., Miriyala K., Grave D. A. (2024). ACS Appl. Energy Mater..

[cit25] Kim J. H., Kim J. H., Kim J. H., Kim Y. K., Lee J. S. (2020). Sol. RRL.

[cit26] Guo Y., Zhang N., Wang X., Qian Q., Zhang S., Li Z., Zou Z. (2017). J. Mater. Chem. A.

[cit27] Grätzel M. (2001). Nature.

[cit28] Piekner Y., Dotan H., Tsyganok A., Malviya K. D., Grave D. A., Kfir O., Rothschild A. (2018). ACS Photonics.

[cit29] Klotz D., Grave D. A., Rothschild A. (2017). Phys. Chem. Chem. Phys..

[cit30] Sinkkonen J., Ruokolainen J., Uotila P., Hovinen A. (1995). Appl. Phys. Lett..

[cit31] Ouellette O., Lesage-Landry A., Scheffel B., Hoogland S., García de Arquer F. P., Sargent E. H. (2019). Adv. Funct. Mater..

[cit32] Segev G., Dotan H., Ellis D. S., Piekner Y., Klotz D., Beeman J. W., Cooper J. K., Grave D. A., Sharp I. D., Rothschild A. (2018). Joule.

[cit33] Segev G., Jiang C. M., Cooper J. K., Eichhorn J., Toma F. M., Sharp I. D. (2018). Energy Environ. Sci..

[cit34] Cvejić Ž., Rakić S., Jankov S., Skuban S., Kapor A. (2009). J. Alloys Compd..

