# Supplementary material for: Quantification of mobile charge carrier yield and transport lengths in ultrathin film light-trapping ZnFe2O4 photoanodes
Source: J Mater Chem A Mater. 2024 Dec 18;13(4):2965–73. doi: 10.1039/d4ta05448b (PMC11653470; doi:10.1039/d4ta05448b)
Supplement: TA-013-D4TA05448B-s001 [file TA-013-D4TA05448B-s001.pdf]

Supplementary Information (SI) for Journal of Materials Chemistry A.
This journal is © The Royal Society of Chemistry 2024

Supporting Information

Quantification of mobile charge carrier yield and transport lengths in ultrathin film light-trapping $ZnFe_2O_4$ photoanodes

Kumaraswamy Miriyala[1,2], Sa'ar Shor Peled[1,2], Dino Klotz[3], and Daniel A. Grave[1,2]*

[1]Department of Materials Engineering, Ben-Gurion University of the Negev, Beer Sheva 8410500, Israel
[2]Ilse Katz Institute for Nanoscale Science and Technology, Ben-Gurion University of the Negev, Beer Sheva 8410500, Israel
[3]International Institute for Carbon-Neutral Energy Research (I2CNER), Kyushu University, Fukuoka, Japan
*Email: dgrave@bgu.ac.il

Ellipsometry Analysis:

To avoid accumulative errors, the Epi-Ag/mica layer was first measured and modelled. Following this, each layer was measured and modelled individually before modelling the full stack.

For the Epi-Ag layer, we employed a model we recently reported.[1] The model parameters are shown in table S1 below. For the Nb:SnO_2 (NTO) layer, one Drude oscillator was used to account for free carriers introduced by the Nb doping, and two Lorentz oscillators to account for above band-gap absorption. For Ti:ZFO, one Tauc-Lorentz oscillator was used for band-edge absorption, followed by five Gaussian oscillators to fully model the layer.

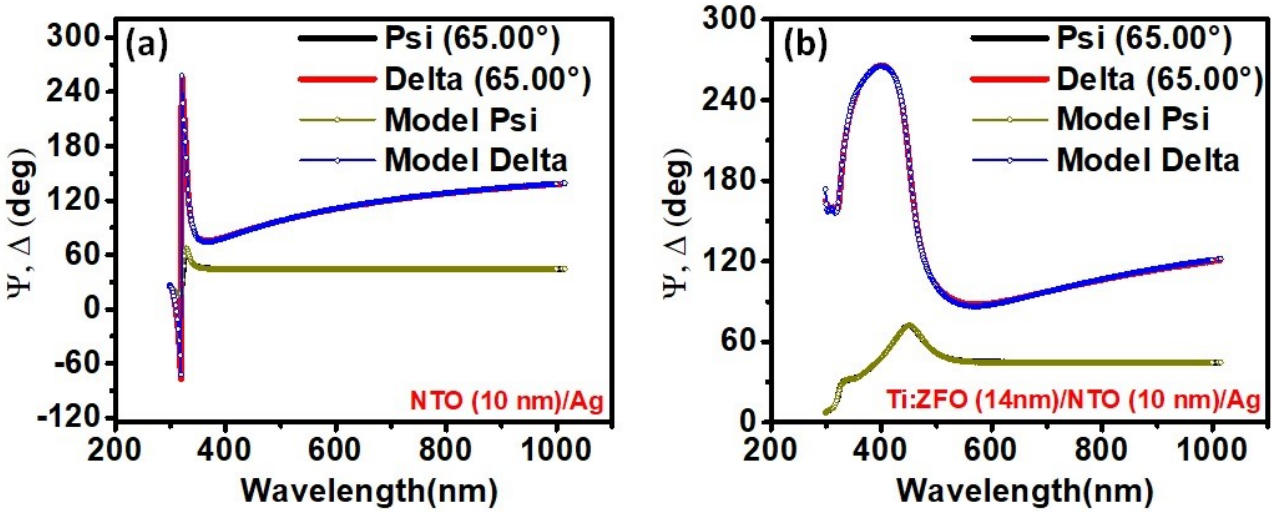

Figure S1. Ellipsometry measurements and models of (a) NTO/Ag and (b) Ti:ZFO/NTO/Ag multilayer stacks

Table S1. Ellipsometer models of Ag, NTO, and Ti:ZFO layers

Layer	Oscillator	Oscillator Parameters
Epi-Ag	Drude	Resistivity (Ω cm)=3.5e-3<br>Scat. Time (fs)=0.255
	PSemi-M0	Amp=0.803<br>Br=0.0418<br>E0=3.917<br>WR=3.7896<br>PR=0.831<br>AR=4.16<br>O2R=0
Nb:SnO_2	Drude	Resistivity (Ω cm)=7.3e-4<br>Scat. Time (fs)=8.001
	Lorentz	Amp=1.037460<br>Br=0.1908<br>En=3.821
	Lorentz	Amp=0.4057<br>Br=0.2507<br>En=4.060
Ti:$ZnFe_2O_4$	Tauc-Lorentz	Amp=0.5973<br>Br=0.182<br>E0=2.476<br>Eg=2.00
	Gaussian 1	Amp=0.1318<br>Br=0.1881<br>En=2.885
	Gaussian 2	Amp=2.6254<br>Br=0.7593<br>En=3.346
	Gaussian 3	Amp=1.5867<br>Br=0.3464<br>En=3.585
	Gaussian 4	Amp=3.8723<br>Br=0.1802<br>En=3.820
	Gaussian 5	Amp=4.6917<br>Br=0.4152<br>En=4.093

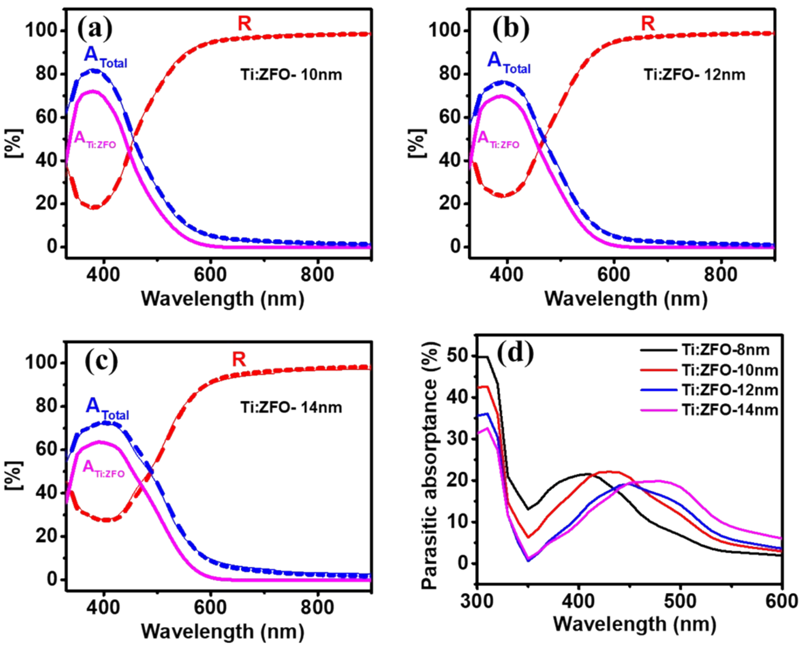

Figure S2: (a)-(c) Simulated and measured reflectance and absorptance of the total device for the 10, 12, and 14nm films. Also shown is the calculated absorptance in the Ti:ZFO active layer alone.(d) The parasitic absorptance in the Ag and NTO layers for all the films.

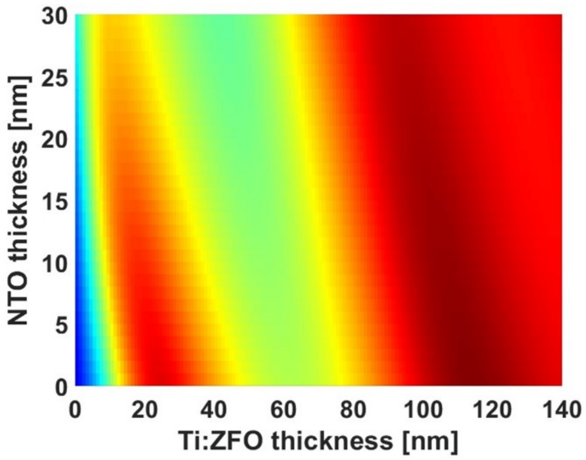

Figure S3. Calculated J_{abs} map in total device including parasitic absorption as a function of NTO and Ti:ZFO thickness

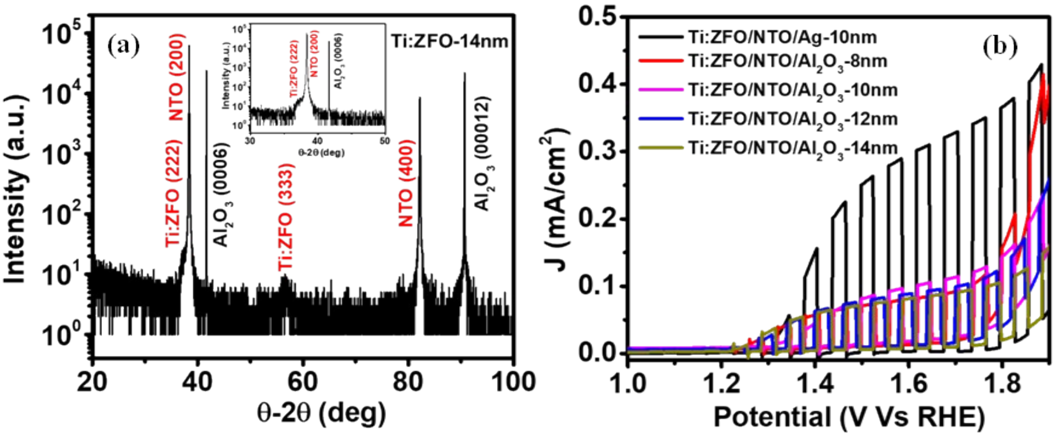

Figure S4: (a) HR-XRD of 14nm epitaxial Ti:ZFO film deposited on NTO/Al_2O_3 substrate. Inset shows enlarged view between 30-50° shows the Ti:ZFO (222) peak along with NTO (200) and Al_2O_3 (0006) reflections. (b) represents the comparison of linear voltammograms of Ti:ZFO-10nm with all other thickness (8-14nm) of Ti:ZFO/NTO/Al_2O_3 films measured under solar simulated light in 1M NaOH solution.

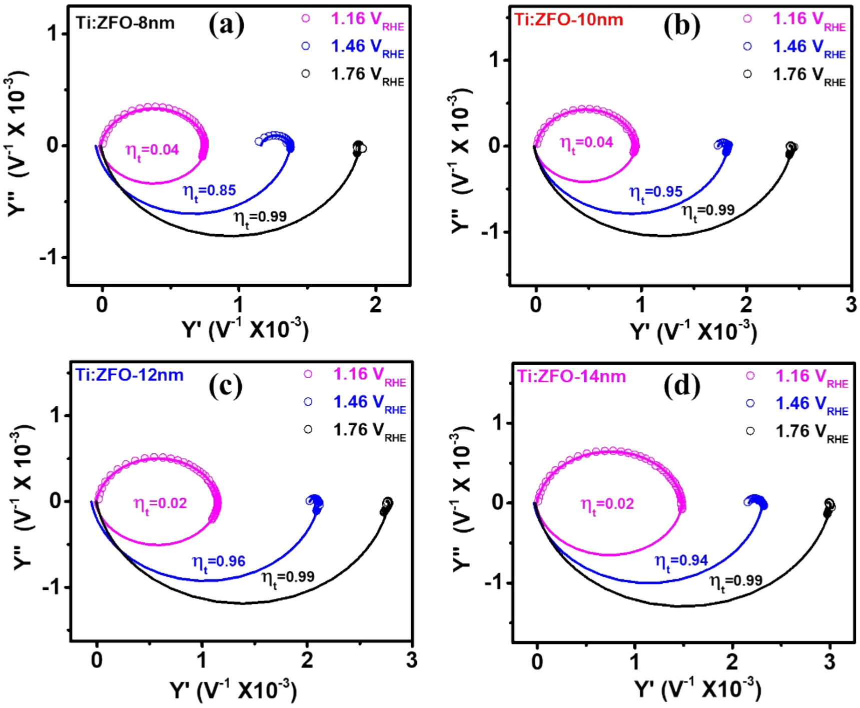

Figure S5: (a)-(d) shows the IMPS spectra of 8, 10, 12 and 14 nm Ti:ZFO films measured at three potentials (1.16, 1.46, 1.76 V_{RHE}) under constant light intensity (100 mW cm^{-2}). Measured data (open circles), fitting (solid line).

Table S2. Charge transfer efficiencies for Ti:ZFO films

Thickness	η_t (%) 1.16 V_{RHE}	η_t (%) 1.46 V_{RHE}	η_t (%) 1.76 V_{RHE}
8nm	4	85	99
10nm	4	94	99
12nm	2	96	99
14nm	2	94	99

Table S3. Charge separation efficiencies for Ti:ZFO films

Thickness	η_{cs} (%) 1.16 V_{RHE}	η_{cs} (%) 1.46 V_{RHE}	η_{cs} (%) 1.76 V_{RHE}
8nm	0.16	1.5	13
10nm	0.17	7.5	11
12nm	0.2	3.4	9.2
14nm	0.3	5.9	8.5

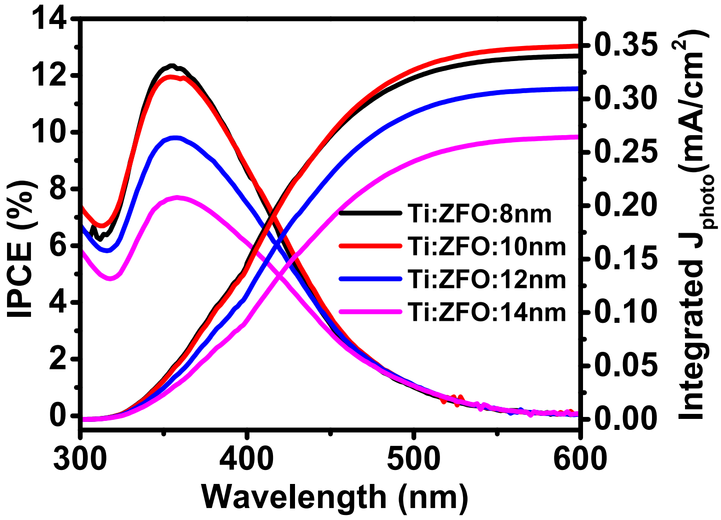

Figure S6: represents the IPCE spectra measured at 1.76 V_{RHE} for 8, 10, 12, and 14 nm Ti: ZFO thin films along with the integration of corresponding IPCE spectra over standard AM 1.5 G solar spectrum (100 mW cm^{-2}).

Table S4. J_{photo} comparison between LSV and IPCE

Thickness	J_{photo} (μA/cm^2) - LSV	J_{photo} (μA/cm^2) - IPCE
8nm	342	340
10nm	345	350
12nm	321	310
14nm	322	264

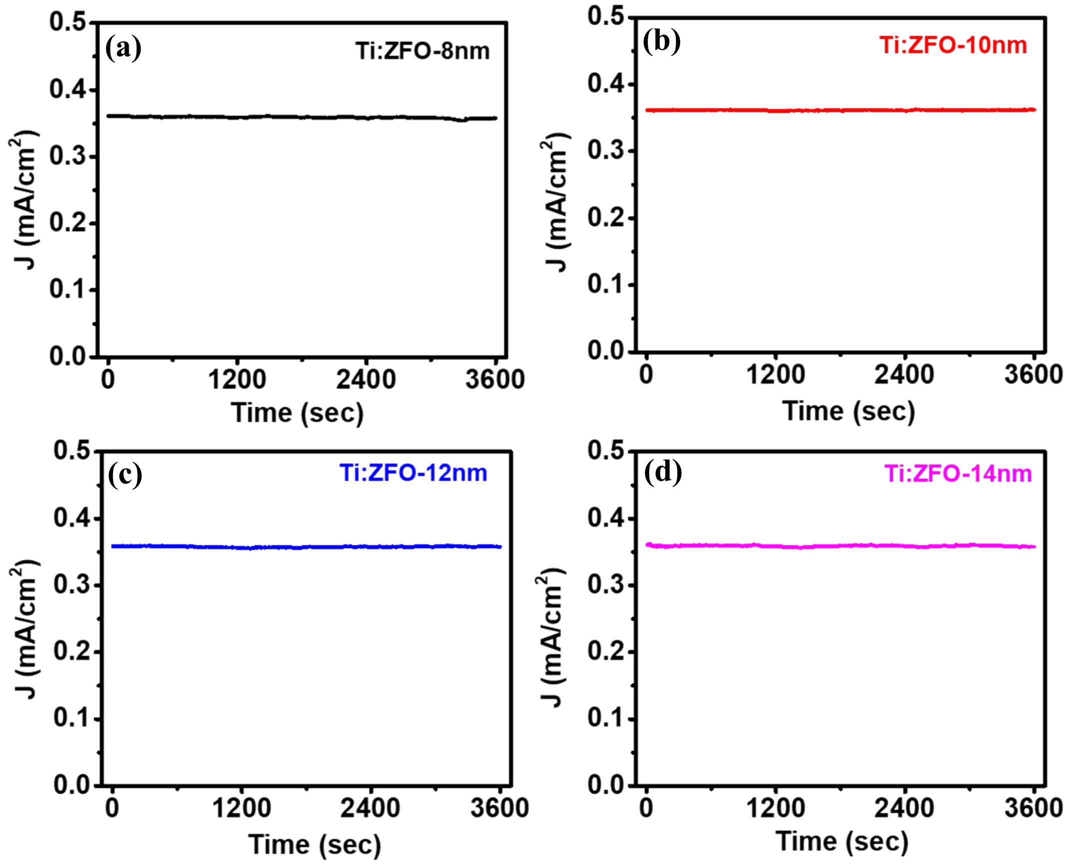

Figure S7: (a)-(d) represents the 1hr stability tests of 8, 10, 12 and 14 nm Ti:ZFO films measured by chronoamperometry technique at a potential of 1.76 V_{RHE} under white light illumination.

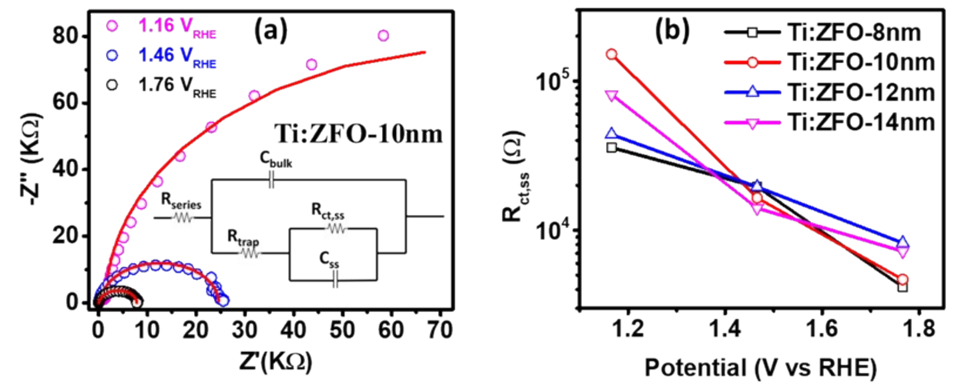

Figure S8: (a) Nyquist plot of Ti:ZFO-10nm film measured at three potentials (1.16, 1.46, 1.76 V_{RHE}) under solar simulated light. The measured response is showed in open circles and fitting curve is showed in solid line format. In inset figure we have shown the equivalent circuit model used for the fitting. (b) comparison plot of charge transfer resistance ($R_{ct,ss}$) for all the thickness over three potentials (1.16, 1.46, 1.76 V_{RHE}).

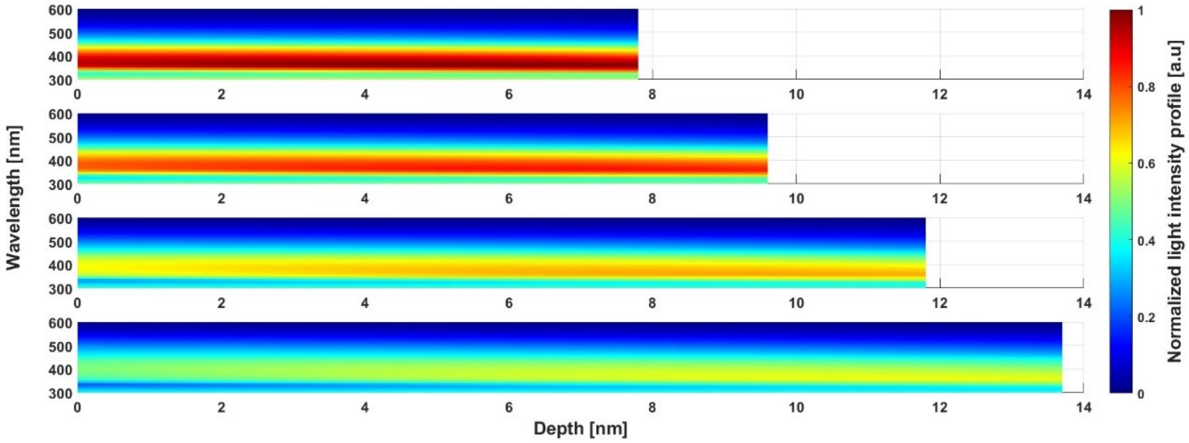

Figure S9. Light intensity profiles for 8, 10, 12 and 14 nm thick films

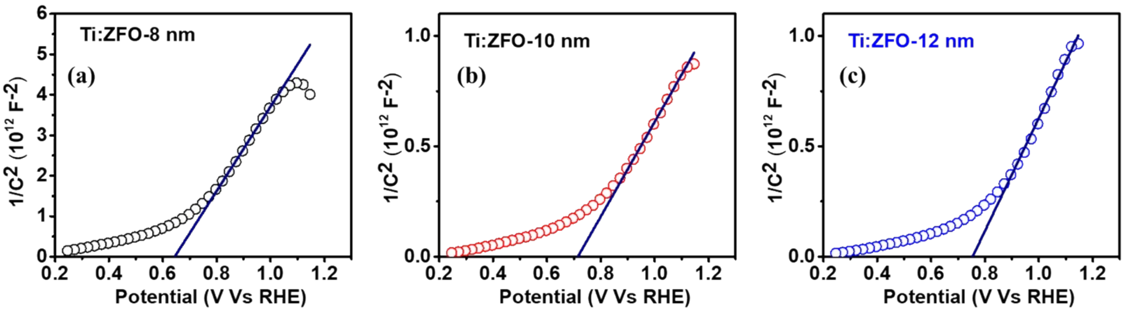

Figure S10. (a)-(c) Mott-Schottky plots of 8,10 and 12nm thick Ti:ZFO films.

Table S5. Extracted values of N_D, W and E_{fb} for Ti:ZFO films from Mott-Schottky plots

Thickness	N_D (cm^{-3})	W at 1.76 V_{RHE} (nm)	E_{fb} (V vs. RHE)
8 nm	1.2 ± 0.1 x 10^{19}	32.1 ± 1.8	0.68±0.07
10 nm	5.2 ±1.2 x 10^{19}	15.2 ± 2.0	0.71±0.01
12 nm	4.29±0.8 x 10^{19}	16. 3± 1.9	0.76 ± 0.01
14 nm	3.5±0.5 x 10^{19}	18.2 ± 1.2	0.74 ± 0.01

References

1 S. Shor Peled, K. Miriyala, A. Rashkovskiy, R. Fishov, V. Gelberg, J. Pelleg and D. A. Grave, *ACS Appl. Mater. Interfaces*, 2023, **15**, 57273–57281.
